# Role of Sirtuins in Modulating Neurodegeneration of the Enteric Nervous System and Central Nervous System

**DOI:** 10.3389/fnins.2020.614331

**Published:** 2020-12-22

**Authors:** Pavithra Chandramowlishwaran, Anitha Vijay, Daniel Abraham, Ge Li, Simon Musyoka Mwangi, Shanthi Srinivasan

**Affiliations:** ^1^Division of Digestive Diseases, Department of Medicine, Emory University School of Medicine, Atlanta, GA, United States; ^2^Research-Gastroenterology, Atlanta Veterans Affairs Health Care System, Decatur, GA, United States; ^3^Department of Veterinary and Biomedical Sciences, The Pennsylvania State University, University Park, PA, United States; ^4^Sidney Kimmel Medical College, Thomas Jefferson University, Philadelphia, PA, United States

**Keywords:** central nervous system, enteric nervous system, gut microbiota, myenteric plexus, neuronal survival, neurodegeneration, sirtuin (SIRT)

## Abstract

Neurodegeneration of the central and enteric nervous systems is a common feature of aging and aging-related diseases, and is accelerated in individuals with metabolic dysfunction including obesity and diabetes. The molecular mechanisms of neurodegeneration in both the CNS and ENS are overlapping. Sirtuins are an important family of histone deacetylases that are important for genome stability, cellular response to stress, and nutrient and hormone sensing. They are activated by calorie restriction (CR) and by the coenzyme, nicotinamide adenine dinucleotide (NAD^+^). Sirtuins, specifically the nuclear SIRT1 and mitochondrial SIRT3, have been shown to have predominantly neuroprotective roles in the CNS while the cytoplasmic sirtuin, SIRT2 is largely associated with neurodegeneration. A systematic study of sirtuins in the ENS and their effect on enteric neuronal growth and survival has not been conducted. Recent studies, however, also link sirtuins with important hormones such as leptin, ghrelin, melatonin, and serotonin which influence many important processes including satiety, mood, circadian rhythm, and gut homeostasis. In this review, we address emerging roles of sirtuins in modulating the metabolic challenges from aging, obesity, and diabetes that lead to neurodegeneration in the ENS and CNS. We also highlight a novel role for sirtuins along the microbiota-gut-brain axis in modulating neurodegeneration.

## The Effects of Age, Obesity, and Diabetes on Neurodegeneration in the CNS and ENS

Neurodegeneration in the CNS is characterized by a progressive loss of distinct groups of neurons in specific regions of the brain, deposition of misfolded proteins in neurons, and alterations in astrocytes (Przedborski et al., 2003; Maragakis and Rothstein, 2006) which results in cognitive dysfunction, loss of synapses, impaired synaptic plasticity, disrupted neuronal signaling, and cell death. The factors that contribute to neuronal stresses in the CNS are aging, neurodegenerative diseases (NDs), comorbidities such as obesity and diabetes, over nutrition via high calorie intake, a lack of physical activity, and genetic background (Popa-Wagner et al., 2020). These stresses and changing physiological demands from oxidative damage, protein aggregation, dietary changes, inflammation, high metabolic demands, are counteracted by cells to maintain cellular, protein, and metabolic homeostasis (Squier, 2001; Uttara et al., 2009). Similar to the CNS, the neurons of the enteric nervous system (ENS) or the “brain within the gut,” are also prone to neurodegeneration. The ENS is a subdivision of the peripheral nervous system and functions independently of the central nervous system (Nezami and Srinivasan, 2010). The ENS is embedded within the walls of the intestine and directly controls gastrointestinal functions. The neurons and glia in the ENS are structurally organized into two interconnected layers, the myenteric and the submucosal plexi. The myenteric plexus, which is located between the circular and longitudinal muscle layers, regulates gastrointestinal motility whereas the submucosal plexus, which is located between the circular muscle and mucosa, regulates secretory activity. In these plexuses, the neuronal cells form groups of interconnected ganglions that are surrounded by glia. The individual ganglia are connected to each other and to the epithelium by neuronal projections (Nezami and Srinivasan, 2010). The ENS, intestinal epithelium, gut microbiota, and immune cells work in harmony together to ensure the proper functioning of the intestine (Walsh and Zemper, 2019). ENS-related neurodegeneration is particularly evident in individuals with aging and neurodegenerative disorders. Moreover, individuals with aging or NDs also experience symptoms related to gastrointestinal dysmotility especially chronic constipation due to loss of enteric neurons in the myenteric plexus leading to ENS dysfunction (Poirier et al., 2016; Rao and Gershon, 2016). Additional stresses from dietary changes and antibiotic treatments can alter the gut microbiota and also influence ENS function (Carabotti et al., 2015). Further understanding of the factors leading to neurodegeneration of the CNS and ENS is critical and can lead to new therapeutic targets.

### Aging Associated Neurodegeneration

Aging is a multifactorial process accompanied by many changes at the cellular, tissue, and organismal level over time and has shown to be a major risk factor for neurodegeneration of the CNS and the ENS (Wade and Cowen, 2004; Hou et al., 2019). Cross-sectional, longitudinal, and quantitative magnetic resonance imaging (MRI) and voxel-based morphometry (VMI) analyses report reduced brain volume and brain atrophy especially in the hippocampus and the prefrontal cortex, in conjunction with a decline in cognitive functioning in older adults (Liu et al., 2003; Terribilli et al., 2011; Ramanoel et al., 2018). *In vitro* studies in rat primary cortical and hippocampal neurons, cultured long term, demonstrate irreparable DNA damage that underlies normal aging leading to proteostasis and cell senescence (Ishikawa and Ishikawa, 2020). This causes a loss of ability to repair tissues, chemokine and cytokine release, low grade inflammation, and results in age-related neurodegeneration. Population-based studies of cognitively unimpaired aged people reported an accumulation of abnormal protein deposits that positively correlated with age (Elobeid et al., 2016). While age-associated neurodegeneration is accompanied by a gradual loss of neurons, rapid progression in behavioral and cognitive changes have been attributed to chronic neurodegenerative diseases (ND) such as Alzheimer's disease (AD) and Parkinson's disease (PD) (Wilson et al., 2010). Along with these functional declines, the cerebral levels of neurotransmitters such as dopamine, acetylcholine, serotonin, and norepinephrine, and neurotrophic factors such as brain-derived neurotrophic factor (BDNF) and nerve growth factor (NGF) are dramatically reduced in aging brains (Vecchio et al., 2018).

**Figure 1:**
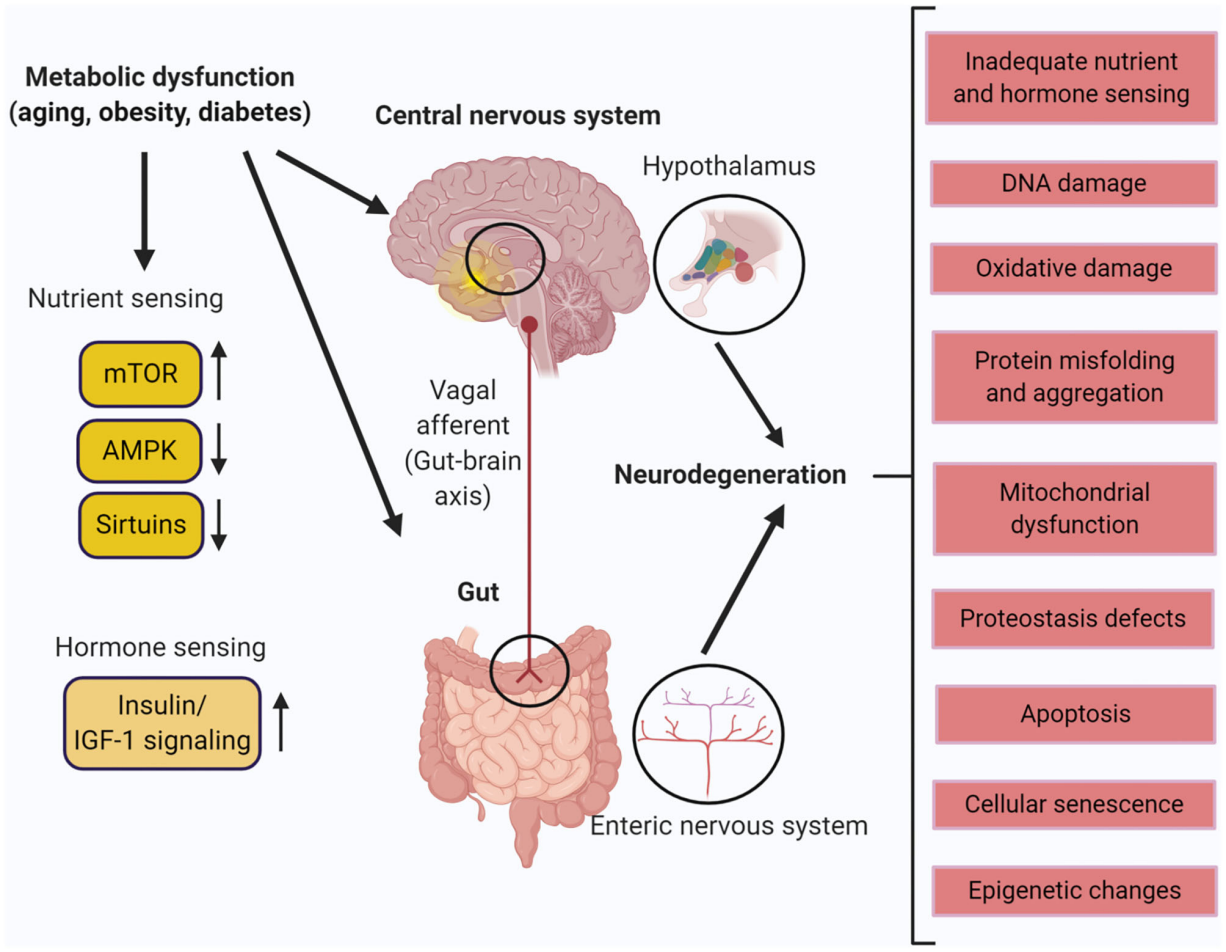
Metabolic dysfunction in the central nervous system (CNS) and the enteric nervous system (ENS). Aging, obesity, and diabetes leads to different molecular and physiological changes that cause neurodegeneration in the hypothalamus of the CNS and the ENS of the gut. Created with BioRender.com.

The impact of aging on the ENS is controversial. Enteric neurons from the colon of human tissues have been shown to decrease in the 4th year of age in both the plexuses with a further loss of 37% of total neuron population between the ages 20–65 (Gomes et al., 1997). Some studies have reported a loss of 50–60% of myenteric neurons has been reported in the aging guts and especially the colon of aging rats (Santer and Baker, 1988; Nezami and Srinivasan, 2010) while other studies contrasted that myenteric neuron numbers in the aging colon remain the same albeit with functional changes (Gamage et al., 2013). Neurotransmitters produced by myenteric neurons and neuronal reflexes mediate late neurogenesis and regulate intestinal motility (Cooke, 2000; Nezami and Srinivasan, 2010). Neurons that produce the choline acetyl transferase (ChAT) and neuronal NOS (nNOS) regulate intestinal motility by stimulating and inhibiting intestinal smooth muscles, respectively (Porter et al., 2002). An imbalance in the equilibrium between the nNOS- and ChAT-producing neurons can alter the ENS architecture and result in an altered bowel motility (Nezami and Srinivasan, 2010). These changes presumably alter the normally well-orchestrated crosstalk between the enteric neurons and glia. Some studies also report a loss of choline acetyltransferase (ChAT) and no changes in neuronal nitric oxide synthase (nNOS)- expressing myenteric neurons (Phillips et al., 2003; Nezami and Srinivasan, 2010) while other studies report the loss of nNOS and ChAT neurons in the myenteric plexus of aging mice (Becker et al., 2018; Sun et al., 2018). In rats, studies have reported a loss of submucosal neurons in the proximal and distal colon in 12 months old animals when compared to 3 month old animals with a greater loss occurring in the distal colon and maximum loss occurring at 24 months of age (Saffrey, 2013). In samples from human colon and ileum, the myenteric ganglia had a wider area overall, with a larger proportion of them with increased gaps within the ganglia and this correlated with increasing age, that might contribute to gut dysmotility seen in older individuals (Hanani et al., 2004). Factors and pathways cumulatively associated with neurodegeneration in the CNS and the ENS with aging (Wyss-Coray, 2016) are diagrammatically represented in Figure 1.

### Neurodegeneration Associated With Metabolic Disorders—Obesity and Diabetes

Obesity and type II diabetes (T2D) have been suggested to accelerate the physiological process of aging (Thorpe and Ferraro, 2004; Kalyani et al., 2017) and obesity is a known risk factor for T2D development (Al-Goblan et al., 2014). In age-dependent and age-independent studies, obesity has been shown to double the risk for mild cognitive impairment, dementia, and AD (Qiu et al., 2009; Hildreth et al., 2012). T2D causes brain atrophy, reduced cerebral glucose metabolism, and insulin resistance in the CNS, and this is also seen in AD (Arnold et al., 2018). Accumulation of misfolded phosphorylated tau and amyloid beta (Arnold et al., 2018) in the brain as well as the islet amyloid polypeptide (amylin) (Raimundo et al., 2020) co-secreted with insulin in the islet beta cells are major pathological features observed in T2D patients who develop AD. As seen in aging, computed tomography demonstrated structural changes in the obese brain such as atrophy in the hippocampus and decreased hippocampal volume (O'Brien et al., 2017). The prefrontal cortex and the hippocampus which are crucial for learning and memory are most vulnerable to obesity-related changes (Bischof and Park, 2015). The hypothalamus controls metabolic homeostasis by sensing nutrients and hormones via autonomic and neuroendocrine signaling to integrate the signals of satiety. Inflammation of the hypothalamus from high fat feeding induces Inhibitor Of Nuclear Factor Kappa-B (IKKβ)/NF-kB-dependent inflammation, changes satiety control, and increases the risk for developing obesity (Timper and Bruning, 2017). Magnetic resonance imaging (MRI) has shown an inverse relationship between Body Mass Index (BMI) as well as diabetes and brain volume, neuron viability, and gliosis in the hypothalamus (Thomas et al., 2019). Obese individuals with higher BMI with no cognitive defects also displayed decreased gray matter, brain atrophy in the frontal lobe, hippocampus and thalamus when compared to non-obese, thus demonstrating extensive neurodegeneration (Stillman et al., 2017). Vascular defects from obesity leading to cognitive decline include lipotoxicity, diabetes impaired insulin metabolism and signaling pathway or defect in glucose transport mechanisms in the brain (Uranga and Keller, 2019). Adipose tissues including white adipose tissue (WAT) are important for metabolism and hormones derived from WAT such as leptin and adiponectin are involved in regulating obesity and diabetes (Stern et al., 2016). Leptin plays a major role in body weight regulation and reducing appetite (Ramos-Lobo and Donato, 2017). Leptin bound to its receptor Ob-Rb has been shown in the cortex and the hippocampus which are major sites of neurodegeneration in aging, AD and PD (McGregor and Harvey, 2018). Another adipokine, adiponectin has been shown to have an opposite effect to leptin in inflammation and insulin resistance, and the ratio of leptin to adiponectin is considered as a marker for developing T2D and obesity (Forny-Germano et al., 2018). High levels of circulating leptin caused by obesity has been shown to lead to leptin resistance in the hypothalamus and is linked to altered metabolism, inflammation, and neurodegeneration in the brain (Forny-Germano et al., 2018). Obesity and T2D also cause gastrointestinal dysmotility and lead to enteric neuronal degeneration (Yarandi and Srinivasan, 2014). Diabetic autonomic neuropathy has been shown to gastrointestinal disturbances including impaired esophageal transit, gastroparesis, and disorganized intestinal motility with constipation and diarrhea (Verrotti et al., 2014). Our studies in obese and overweight human subjects and in mice fed a high fat diet have demonstrated increased pyroptosis in nitrergic neurons, delayed colonic transit, and impaired electric field stimulation–induced colonic relaxation responses (Ye et al., 2020). In mouse Other studies in the ENS using mice models of high fat diet and obesity induced diabetic neuropathy reported a reduction in hormones such as ghrelin, cholecystokinin (CCK), and leptin levels; and inhibitory neurons expressing nitric oxide synthase (nNOS), vasoactive intestinal peptide (VIP), neuropeptide Y (NPY), and galanin as well as their expression levels (Chandrasekharan and Srinivasan, 2007; Li et al., 2011; Voukali et al., 2011; Stenkamp-Strahm et al., 2015). The neuronal changes characterized by the loss of important neurotransmitters and hormones resulted in altered gastric emptying, diarrhea and constipation that is characteristic of enteric neurodegeneration (Stenkamp-Strahm et al., 2015). Moreover, these alterations could be a compensatory mechanism to increase satiety and decrease food intake to balance weight gain in diet induced obese mice (Coll et al., 2007). A major orchestrator of pathways in response to stresses caused by age- and metabolism-associated neurodegeneration are the sirtuins (Duan, 2013). Factors and pathways cumulatively associated with neurodegeneration in the CNS and the ENS with metabolic dysfunction are summarized in Figure 1.

## Function and Distribution of Sirtuins

### Localization, Activators, and Substrates of Sirtuins

Silent information regulator (Sirtuins) are a family of class III histone deacetylases with a conserved catalytic domain core of 275 amino acids (Houtkooper et al., 2012). The subcellular localization, enzymatic activities, transcriptional substrates, functions, and activators of sirtuins are briefly explained in Table 1. As cooperative sensors and regulators of nutrients and energy metabolism in response to changes in diet and stress, they require NAD^+^ for their enzymatic activity (Anderson et al., 2017). Energy deficits by calorie restriction or cellular stressors increase NAD^+^ levels and activate sirtuins (Guarente, 2013).

**Table 1:** Overview of sirtuin localization, activity, substrates, functions, and activators.

**Sirtuin**	**Specific examples relevant to metabolism**
Localization	Sirtuins are present in all subcellular compartments and differ in their substrate specificities (Houtkooper et al., 2012). SIRT1, SIRT6, and SIRT7 are predominantly nuclear but also detected at lower levels in cytosol, membrane, and the cytoskeleton. SIRT2 resides in the cytoplasm (Houtkooper et al., 2012) though it is also found in the nucleus and the cell membrane (North and Verdin, 2007). SIRT3, SIRT4, and SIRT5 predominantly localize to the mitochondria although they are also found in the nucleus and the cytoplasm (Houtkooper et al., 2012).
Enzymatic activity	SIRT1, SIRT2, SIRT3, SIRT5, and SIRT7 predominantly deacetylate histone and non-histone proteins; SIRT4 and SIRT6 act as mono-ADP-ribosyl transferases (Canto et al., 2013).
Functions	SIRT1 and SIRT3—Neuronal protection and cell survival, DNA repair, chromatin remodeling, neuronal differentiation, apoptosis, energy and metabolic homeostasis, mitochondrial biogenesis, autophagy, glucose production and insulin secretion, lipid homeostasis, anti-inflammation (Yamamoto et al., 2007; Duan, 2013; Giblin et al., 2014). SIRT2—cell cycle regulation, modulation of microtubule deacetylation and myelination, tumorigenesis, neurodegeneration (Yamamoto et al., 2007; Gomes et al., 2015). SIRT4—insulin secretion, cell cycle regulation (Yamamoto et al., 2007). SIRT5—Mitochondrial metabolism, urea cycle (Yamamoto et al., 2007). SIRT6—glucose homeostasis, genome stability, DNA repair, anti-inflammation (Yamamoto et al., 2007; Zhong and Mostoslavsky, 2010). SIRT7—rDNA transcription (Wu et al., 2018).
Histone targets	H1, H3, H4 (H1K26, H1K9, H3K9, H3K56, H3K14, H4K16) by SIRT1; H4K16 by SIRT2; H3, H4 (H3K9, H4K16) by SIRT3; H2B, H3 (H2BK12, H3K9, H3K56) by SIRT6; and H2A, H2B, H3 (H3K18) by SIRT7 (Jing and Lin, 2015).
Non-histone targets	Transcriptional regulators (Martinez-Redondo and Vaquero, 2013; Jing and Lin, 2015) related to: •Stress - p53, Nuclear Factor kappa B (NF-κB), Forkhead Box (FoxO), Superoxide dismutase 2 (SOD2), Poly (ADP-ribose) polymerase (PARP), target of rapamycin (TOR) kinase (TORC2), bcl-2-like protein 4 (Bax), leucine zippers - c-Fos and c-Jun, Uncoupling Protein 2 (UCP2), Heat shock factor 1 (HSF1), b-catenin, E2F Transcription Factor 1 (E2F1), Period Circadian Regulator 2 (PER2), Circadian Locomotor Output Cycles Kaput (CLOCK) •Metabolism - Peroxisome proliferator-activated receptor gamma coactivator 1-alpha (PGC-1α), Liver X receptor (LXR), Farnesoid X receptor (FXR) •DNA repair - Ku70, Peroxisome proliferator-activated receptor gamma (PPARγ) •Structural protein - α-tubulin •Chromatin remodeling - p300, MOF
Agonists or stimulators	Resveratrol [activated SIRT1 and reduced signs of aging without changing the expression patterns of other sirtuins (Borra et al., 2005)], SRT1720 (Huynh et al., 2013) and oxazolo[4,5-b] pyridines (Bemis et al., 2009) [activated SIRT1 to treat diabetes and insulin resistance in mice (Bemis et al., 2009)], pyrrolo[3,2-b]quinoxalines [promoted SIRT1, SIRT2, and SIRT3-dependent anti-inflammatory properties *in vitro* (Villalba and Alcain, 2012)], and honokiol [activated SIRT3 and counteracted oxidative stress and mitochondrial damage in AD and diabetes studies (Ramesh et al., 2018; Zheng et al., 2018)].

### Distribution of Sirtuins in the CNS and the ENS

All of the seven sirtuins are ubiquitously expressed in all human tissues (Yamamoto et al., 2007). Mass spectrometry and semi-quantitative studies have shown that all the sirtuins are expressed in the human and non-human brain and small intestine (Sidorova-Darmos et al., 2014; Jayasena et al., 2016). In the brain, SIRT1 (110 KDa) and SIRT2 (37 KDa) are the most abundant and widely expressed sirtuin subtypes. SIRT1 expression is highest in the neurons of the cerebellum, hippocampus, and the hypothalamus and lowest in the spinal cord. SIRT2 is highest in the spinal cord and brain stem and is also highly expressed in the cortex, frontal lobe, hippocampus, striatum, and cerebellum. The mitochondrial sirtuins, SIRT3, SIRT4, SIRT5, are also expressed in different regions of the brain, but at lower levels than SIRT1 and SIRT2. In the brain, SIRT6 and SIRT7 are expressed at the lowest levels compared to other sirtuins. All the sirtuins, except for SIRT7, are expressed at lower levels in the small intestine than is detected in the brain. SIRT7 is the most highly expressed sirtuin in the small intestine with 10-fold higher expression than in the brain. Considering that subcellular localization of sirtuins are cell type dependent, more sampling across different cell lines and tissue types can provide information about the anatomical contribution of the lesser abundant sirtuins. Recent studies have shown that both SIRT1 and SIRT3 are expressed by neurons of the ENS (Lakhan and Kirchgessner, 2011; Bubenheimer et al., 2016). However, the role of sirtuins in ENS neurodegeneration remains unknown.

## Role of Sirtuins in Modulating Neurodegeneration in the CNS

### Role of Sirtuins in Modulating Neurodegeneration Associated With Aging and Neurodegenerative Diseases

Aging leads to damage of cellular organelles and accumulation of proteins that causes an imbalance in cellular homeostasis and accelerates neurodegeneration (Castelli et al., 2019). SIRT1 and SIRT6 levels increase and decrease respectively with age respectively despite similarities in cellular localization and their role in increasing lifespan (Lee et al., 2019). A reduction in SIRT1 activity was reported in post-mortem brain tissue of PD patients (Singh et al., 2017). SIRT1 has shown to be universally involved in multiple pathways associated with stress related to energy homeostasis and metabolism through epigenetic regulation and transcriptional modulation whereas SIRT6 is important for glucose metabolism and exerts neuroprotection from DNA damage (Ramadori et al., 2008; Zhong and Mostoslavsky, 2010). Overexpression of brain-specific SIRT1, ubiquitous overexpression of SIRT6, calorie restriction, or resveratrol, extended lifespan and prevented experimental AD amyloid neuropathology (Giblin et al., 2014). Resveratrol has been shown to inhibit the activity of a serine/threonine kinase called mammalian target of rapamycin (mTOR) which contrasts with nicotinamide, a SIRT1 antagonist enhanced mTOR activity and reduced age-induced autophagy (Ghosh et al., 2010). In neurons comprising of non-dividing cells, SIRT1 has been shown to foster DNA repair during double strand breaks and protect against genomic instability caused by aging (Oberdoerffer et al., 2008). Studies in rat brain, kidney, liver, and fat pad tissues showed that SIRT1 induced by calorie restriction maintained a DNA repair factor, Ku70 in a deacetylated state to sequester Bax from the mitochondria to attenuate apoptosis, thus shifting the balance from cell death toward cell survival (Amsel et al., 2008). SIRT1 and in some cases, SIRT2 and the signaling pathways of insulin and insulin-like growth factor-I (IGF-I), bidirectionally regulate each other (Sansone et al., 2013). IGF-1 is an important growth factor that has been shown to be important for neurogenesis and cell survival of neurons as well as inhibition of apoptosis during postnatal to adult stages (Nieto-Estevez et al., 2016). IGF-1 declines with age in the brains of humans and rodents, and treatment with IGF-1 agonists in preclinical models of AD and PD have shown to improve neuronal survival (Nieto-Estevez et al., 2016). Notably, SIRT1 deacetylates insulin receptor substrate 2 (IRS-2), a substrate protein for IGF-1 and activates Akt, an insulin receptor target of IGF (Sansone et al., 2013), highlighting SIRT1 importance in modulating IGF-1 signaling.

Overexpression of SIRT1 and the addition of resveratrol has shown to provide neuroprotective effects in various animal models of AD by reducing amyloid plaque formation and neurofibrillary tau pathology (Chen et al., 2005; Qin et al., 2006; Kim et al., 2007; Green et al., 2008; Karuppagounder et al., 2009; Min et al., 2010; Vingtdeux et al., 2010). SIRT1 was shown to target ADAM10, a retinoic acid receptor β target and induce Notch receptor cleavage to promote non-amyloidogenic processing of amyloid precursor protein (APP), thereby promoting neurogenesis (Donmez et al., 2010). Overexpression of SIRT1 protected SH-SY5Y neuroblastoma cells from toxin induced cell death by down-regulating NF-κB and cPARP-1 and reducing phospho-α-synuclein aggregates (Singh et al., 2017). Resveratrol acting via SIRT1/PGC-1α significantly protected dopaminergic neurons in the MPTP mouse model of PD (Mudo et al., 2012). Interestingly in other studies, SIRT1 failed to protect tyrosine hydroxylase (TH)-positive dopaminergic neuronal damage induced by MPTP (Kakefuda et al., 2009). SIRT2 inhibition was shown to reduce Aβ production and improved cognitive performance and microtubule assembly favoring cell survival (Biella et al., 2016; Silva et al., 2017). SIRT3 expression was decreased in AD patient's cerebral cortex and its dysfunction led to p53-mediated mitochondrial and neuronal damage in AD (Lee et al., 2018). Patients with AD showed a reduction in the expression of SIRT6. Increased signs of DNA damage, cell death, and hyperphosphorylated Tau, all features of neurodegenerative diseases, were observed in SIRT6-deficient mice brain (Kaluski et al., 2017) indicating the importance of SIRT6 regulation of DNA repair and maintenance of genomic stability to keep the brain healthy (Giblin et al., 2014; Kugel and Mostoslavsky, 2014). SIRT2 has largely been found as detrimental in several neurodegenerative disorders (Gomes et al., 2015). Polymorphisms in a SIRT2 intron increased susceptibility to AD and its knockout and inhibition studies improved outcomes in a PD model by reducing cytoskeletal pathology and increasing autophagy (Biella et al., 2016; Guan et al., 2016). SIRT2 inhibition may have beneficial effects for PD by rescuing α-synuclein mediated toxicity (Outeiro et al., 2007; de Oliveira et al., 2017). SIRT3 has been demonstrated to protect cortical neurons from various types of stress by increasing mitochondrial antioxidant capacity (Cheng et al., 2016). Mice with SIRT3 deletion was shown to have reduced neuron number, synaptic plasticity, and poor remote memory, thereby dramatically increasing neuronal vulnerability (Kim et al., 2011; Dai et al., 2014). SIRT3 and SIRT5 have the largest protective effects on neurons of the nigrostriatal pathway within the brain (Liu et al., 2015a,b). SIRT5 displays a protective role against MPTP-induced nigrostriatal dopaminergic degeneration by preserving mitochondrial antioxidant capacity (Liu et al., 2015b). Resveratrol and another polyphenol quercetin in mice models were shown to prevent motor neuron degeneration and polyglutamine-induced cell death in striatal neurons characteristic of motor neuron disorders such as amyotrophic lateral sclerosis (ALS), spinal muscular atrophy (SMA), and Huntington Disease (HD), respectively (Bhullar and Rupasinghe, 2013; Lazo-Gomez and Tapia, 2017). SIRT6 and SIRT7 were shown to deacetylate the nucleolar protein, nucleophosmin (NPM1) that is involved in DNA repair to regulate aging (Wu et al., 2018).

### Role of Sirtuins in Modulating Neurodegeneration Associated With Obesity and Diabetes

**Figure 2:**
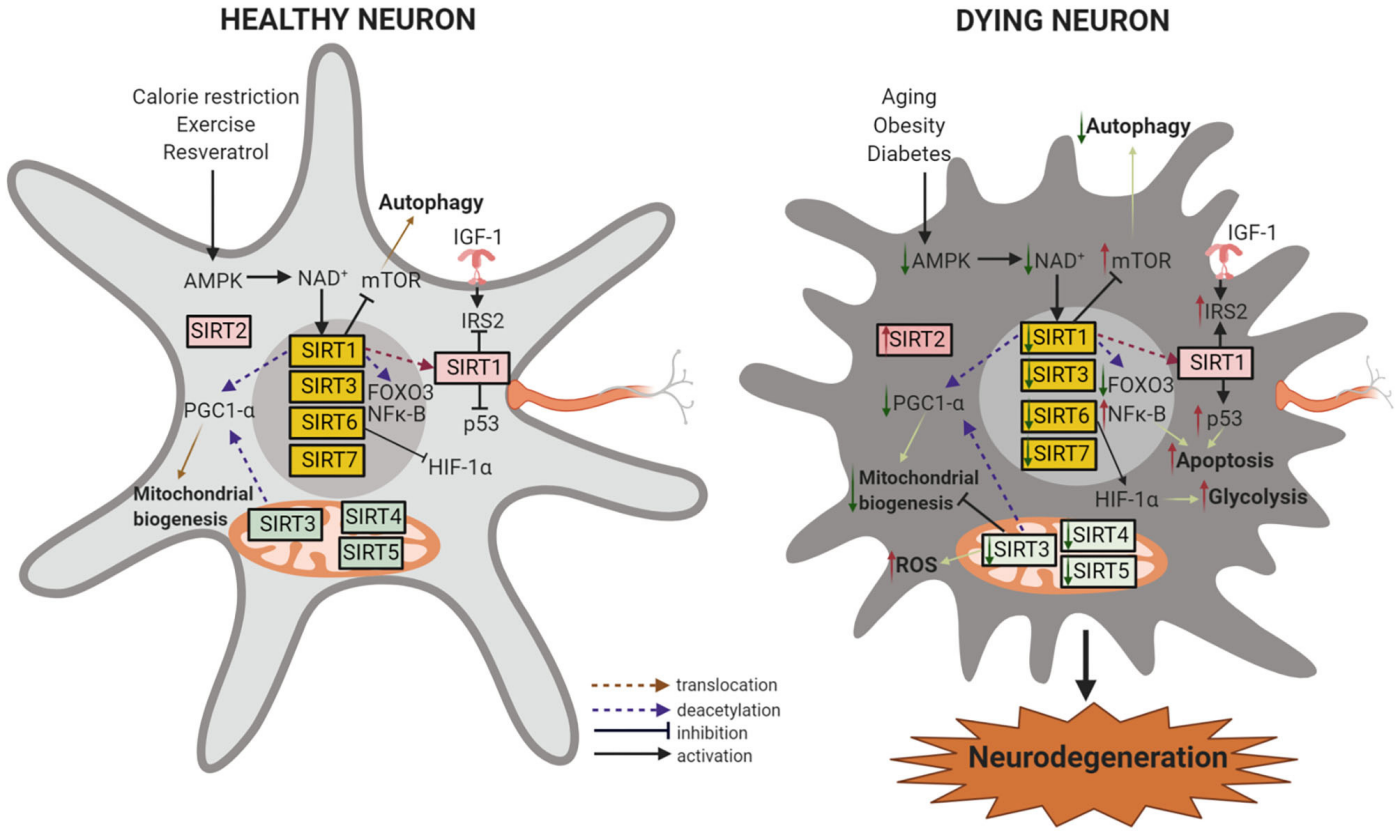
Sirtuins in neuroprotection and neurodegeneration. In healthy neurons, downregulation of IGF-1 and activation of SIRT1 by the availability of NAD^+^ induces the activation of FOXO transcription factors and the transcription of antioxidant genes in the nucleus. SIRT1 and SIRT3 activation by calorie restriction (CR) or by resveratrol also leads to PGC-1α modulation with improved mitochondrial function and decreased oxidative stress. PGC-1α and FOXOs can be directly activated through AMPK-dependent phosphorylation. SIRT1 or SIRT3 activation or SIRT2 inhibition can activate autophagy, leading to neuroprotection. SIRT4 and SIRT5 modulation of fatty acid oxidation and reducing oxidative stress contributes to mitochondrial homeostasis, and SIRT7 regulates nuclear encoded mitochondrial genes. SIRT6 represses the recruitment of HIF-1α to its target gene promoter and inhibits glycolysis and increases mitochondrial respiration. During aging, obesity, and diabetes, the reduced availability of NAD^+^ causes decreased AMPK, SIRT1, and SIRT3 levels which in turn decreases the stimulatory effect of PGC-1α on mitochondrial biogenesis. Decreased SIRT1 reduces mTOR inhibition and reduces autophagy and decreases cell viability. SIRT1 can no longer suppress IGF-1, NF-κB, or p53, acetylates and stabilizes p53, and causes inflammation and apoptosis. Low levels of SIRT4 increases glutamine reflux, dysregulates insulin sensitivity, glucose metabolism, and fatty acid oxidation. SIRT5 and SIRT6 deficiency reduces ATP levels in the mitochondria. Moreover, low levels of SIRT6 leads to increased HIF-1α and results in increased glucose uptake and glycolysis. On the other hand, SIRT2 (or its isoforms) accumulate with age and promote cell death by deacetylating Foxo3a and upregulating a pro-apoptotic factor, Bim. These processes progressively lead to neuronal degeneration and cell death. Created with BioRender.com.

Increased SIRT1 expression in dorsal root ganglion (DRG) neurons was shown to rescue mice from peripheral neuropathy induced by a high fat diet (HFD) (Chandrasekaran et al., 2019). SIRT1 is also regulated by the hypothalamus/pituitary axis that receives inputs related to nutrients and adiposity (Toorie and Nillni, 2014). SIRT1 inhibition in the hypothalamus, via the acetylation of FOXO1, increased neurons that express pro-opiomelanocortin and agouti-related peptide. These neurons produce satiety peptides to inhibit food intake after feeding and increase food intake in response to fasting and CR, respectively. This resulted in reduced feeding and body weight gain (Dietrich et al., 2010). This established the role of SIRT1 as an important regulator of nutrient sensing in the neural circuits that govern central and peripheral networks. SIRT3 deletion in the hippocampus of mice fed a high fat diet was shown to cause oxidative stress and impaired cognition (Tyagi et al., 2018). This was alleviated by SIRT3-mediated aerobic interval training that upregulated the antioxidant manganese superoxide dismutase (MnSOD) and inhibited neuronal apoptosis (Shi et al., 2018). SIRT6 and SIRT7 have been shown to be important for glucose production and metabolism. Studies in SIRT6 deleted mice have shown that SIRT6 deacetylates histone 3 lysine 9 (H3K9) to repress hypoxia-inducible factor, HIF-1α, in the promoter of Glucose transporter type1 (GLUT1) and Pyruvate Dehydrogenase Kinase, Isoenzyme 1 (PDK1) enzymes to facilitate glucose metabolism (Zhong et al., 2010). Knockout studies have shown that SIRT7 is an epigenetic modulator of glucose metabolism that regulates ribosomal biogenesis and promotes mitochondrial biogenesis via PRMT6 methylation and connects it to glucose availability in an AMPK dependent manner (Yan et al., 2018). These studies demonstrate the important functions carried out by SIRT6 and SIRT7 to regulate glucose homeostasis. The pathways relevant to neurodegeneration and modulated by sirtuins are summarized in Figure 2.

## Role Of Sirtuins in Regulation of Neurodegeneration in the ENS

**Figure 3:**
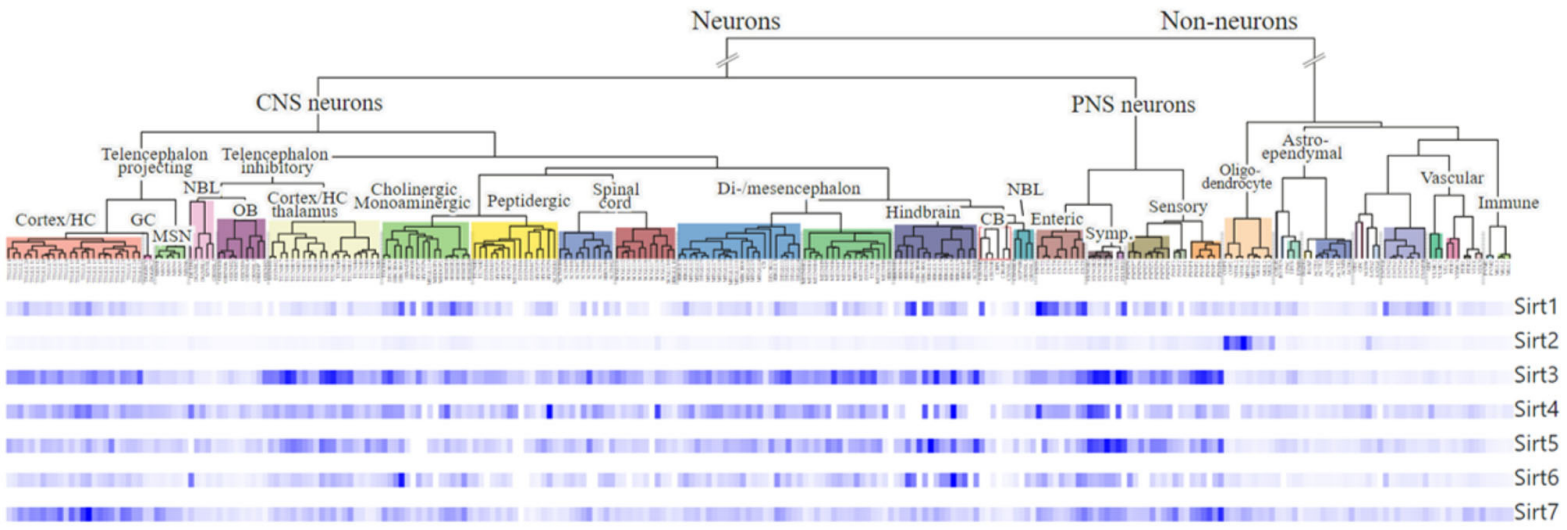
Expression and cellular localization of sirtuins in the neurons of the mouse CNS and the ENS. Dendrogram showing gene expression of all the seven sirtuins in cell populations generated by single-cell RNA sequencing (scRNA-seq) in the central nervous system (CNS) neurons and the enteric neurons in the peripheral nervous system (PNS) using data from Zeisel et al. (2018). The plots were generated using the online database, Mouse Brain Atlas http://mousebrain.org/genesearch.html.

The ENS develops from enteric neural crest cells, a multipotent cell population that originates in the neural tube and migrates across the embryo to reach the developing intestine, where it proliferates and differentiates into enteric neurons and glia (Nagy and Goldstein, 2017). These neural progenitors eventually differentiate into several distinct neuronal subtypes that eventually comprise both the myenteric and submucosal plexi (Furness, 2012). Comparative studies between the intestines of young (3 months old) and old (>24 months) mice (*n* = 6) has shown that aging reduced the number of intestinal stem cells (ISCs) *in vivo* as well as the formation of intestinal organoids from the ISCs *ex vivo* that gives rise to differentiated cells of the gut (Igarashi et al., 2019). The plexuses are areas that are vulnerable to neurodegeneration from aging and high-fat or high sugar induced diets (Lakhan and Kirchgessner, 2011; Stenkamp-Strahm et al., 2015; McMenamin et al., 2018). Sirtuins are widely expressed in the gut (Figure 3, Zeisel et al., 2018) and neurons in the murine colon show immunoreactivity to SIRT1 where they localize to the nucleus, in the myenteric plexus (Lakhan and Kirchgessner, 2011). A knockout of SIRT1 in the gut of mice was reported to increase gastric emptying and intestinal contraction with suppressed villous apoptosis and increased crypt proliferation (Wang et al., 2012). This could indicate an altered cholinergic neuronal function. In the same study, the genes ghrelin and Period Circadian Clock 2 Gene, Per2, which regulate food intake and circadian rhythm respectively (Yannielli et al., 2007; Kim et al., 2018), were also found to be increased in the stomach and hypothalamus, implying a role for SIRT1 in regulating GI functions controlled by the circadian systems. Treatment of aged mice with SIRT1-dependent NAD^+^ precursor, nicotinamide riboside restored ISC number and its functional defects in aged mice *in vivo* (Igarashi et al., 2019) but this was blocked by SIRT1 inhibitor EX527, suggesting a role for SIRT1 activators or precursors in maintaining the intestine during aging. Similar to the role of astrocytes in the CNS, enteric glia modulates the ENS by regulating motility and secretion by sensing neuronal reflexes by virtue of its plasticity (Gulbransen and Christofi, 2018). The glia is also important for epithelial health, and ablation of glia in transgenic mice has shown to cause alterations in motor and mucosal activity, resulting in intestinal inflammation, myenteric degeneration, hemorrhage, and necrosis (Aube et al., 2006). Obesity in the gut is characterized by persistent low-grade inflammation with alterations in gut motility (Hotamisligil, 2006). Experimental data show that gut inflammation, even if mild, could lead to persistent changes in GI nerve and smooth muscle function, resulting in dysmotility, hypersensitivity, and dysfunction (Mawe et al., 2009; Lakhan and Kirchgessner, 2010). Thus, the breakdown of mucosal barrier function as observed in obesity could cause alterations in the patterns of gut motility, abnormal secretion, and changes in visceral sensation that contributes to gastrointestinal symptoms. Whether the changes in GI motility observed in many obese patients are due to inflammation-related changes in the properties of enteric neurons is yet to be explored. Intestinal epithelium-specific knockout of SIRT1 in aged mice induced spontaneous inflammation and tissue damage in the colon and increased their susceptibility to colitis (Wellman et al., 2017). Increased proinflammatory cytokines and leukocyte infiltration, decreased colon lengths, elevated levels of LPS, and increased expression levels of anti-microbial proteins was observed in the SIRT1 KO mice compared to their age-matched controls. SIRT1 induced by resveratrol administration to rats has shown to be protective against acute intestinal inflammation from colitis by downregulating inflammation via NF-κB (Larrosa et al., 2009; Hofseth et al., 2010). In an experimental model of ileitis, oral administration of resveratrol increased the survival of resveratrol-treated mice after exposure to *T. gondii*, decreased mRNA expression of pro-inflammatory cytokine—IL-6, and increased the mRNA expression of anti-inflammatory cytokine—IL-10 in the ileum, compared to the control group (Bereswill et al., 2010). These studies highlight SIRT1 as a potential target in inflammatory diseases of the intestine. Studies in mice and cell cultures have shown that SIRT3 protects cortical and dopaminergic neurons from oxidative stress by regulating mitochondrial homeostasis (Kim et al., 2011; Dai et al., 2014; Shi et al., 2017). Unlike its protective role in the CNS, a SIRT3 knockout in mice exposed to dinitrobenzene sulfonic acid mode of colonic inflammation was shown to not have any effects in counteracting oxidative stress or the susceptibility of myenteric neurons to inflammation (Bubenheimer et al., 2016). Further research is required to explore the role of sirtuin proteins in enteric neurobiology during normal and inflamed states.

## Role of Sirtuins as Modulators of Gut Microbiota Along the Microbiota-Gut-Brain Axis

The gut microbiota regulates many metabolic processes in addition to host energy homeostasis by taking part in the gut–brain crosstalk, a complex bidirectional communication system. This is mediated by gut microbiota produced signaling molecules like short-chain fatty acids (SCFAs: acetate, butyrate and propionate), lipopolysaccharide (LPS), 5-hydroxytryptamine (5-HT), biogenic amines (dopamine, norepinephrine), glutamate and γ-aminobutyric acid (GABA) (Nicholson et al., 2012; O'Mahony et al., 2015; Koh et al., 2016; Mazzoli and Pessione, 2016; Bhattarai et al., 2017; Sudo, 2019). The gut microbiota metabolites affect brain activity either through blood circulation or acting via vagus nerve afferent fibers, while vagal efferent fibers regulate gut permeability and inflammation influencing gut functions (Bonaz et al., 2018). Enteroendocrine cells (EECs) are in direct contact with the luminal contents and mediate the communication between gut microbiota and enteric innervations. They produce hormones and peptides including serotonin, ghrelin, cholecystokinin, glucagon-like peptide-1 (GLP-1), peptide YY (PYY) and pancreatic polypeptide whose receptors are expressed in gut enteric neurons, vagal afferents, brain stem, and hypothalamus (De Silva and Bloom, 2012; Richards et al., 2014). EECs maintain gut homeostasis by regulating food intake and insulin secretion (Gribble and Reimann, 2016). SCFA stimulate the secretion of the leptin, GLP-1, and peptide YY(3–36), and lower body weight thereby contributing to gut-brain activation (Xiong et al., 2004; Tazoe et al., 2008; Tolhurst et al., 2012). The gut-brain bidirectional communication happens largely through the ENS which along with commensal microflora and immune cells, plays an important role in regulating intestinal epithelial barrier function (Snoek et al., 2010). Dysbiosis, an imbalance in the gut microbial community is linked to several metabolic diseases such as obesity, type-2 diabetes mellitus and inflammatory bowel diseases (Castaner et al., 2018; Zuo and Ng, 2018; Sharma and Tripathi, 2019). It is often associated with a reduction in the Bacteroidetes:Firmicutes ratio and increased gut permeability (Tremaroli and Backhed, 2012; Zuo and Ng, 2018; Sharma and Tripathi, 2019) with low-grade gut inflammation. Considerable shifts in human gut microbiota composition have been observed in several CNS disorders including neurodegeneration (Fung et al., 2017). Gut dysbiosis play an important role in modulating the gut–brain axis. An impaired gut barrier facilitate entry of bacterial endotoxins like LPS into the blood circulation, elicit inflammatory response, causing metabolic endotoxemia that eventually leads to insulin resistance and weight gain (van Olden et al., 2015). This impairment can also affect the blood brain barrier promoting neuro-inflammation and neurodegeneration including anxiety and depression (Liu, 2017).

Over recent years, accumulating evidence has suggested the role of sirtuins in obesity, diabetes, and various age-related neurodegenerative diseases by modulating gut microbiota at times, implicating the importance of gut-brain axis connections. Much of the studies involving sirtuins and gut microbiota have been done using resveratrol, which activates SIRT1. Resveratrol is thought to possess antibacterial activity against opportunistic pathogens of the digestive tract like *Escherichia coli, Salmonella enterica*, and *Enterococcus faecalis* (Paulo et al., 2010), thereby contributing to maintenance of normal gut bacterial species. The epithelial barrier integrity and function is regulated by resveratrol by increasing the expression of intestinal tight junction proteins such as tight junction protein 2, occludin, and claudin 4 (Etxeberria et al., 2015; Ling et al., 2016; Wang et al., 2016). Resveratrol up-regulated SIRT1 display anti-inflammatory role in the gut by decreasing immune responses (Th1-type) and preventing bacterial translocation by maintaining gut barrier function (Bereswill et al., 2010), which is compromised in obesity (Cani and Delzenne, 2010). Resveratrol functions by modulating the composition of the gut microbiota (Chen et al., 2016; Komaroff, 2017). Mice studies have suggested that resveratrol can influence the relation between gut microbiota, diet, and obesity (Clarke et al., 2012; Komaroff, 2017) either by changing the expression of genes involved in central regulation of body weight homeostasis like fasting-induced adipose factor (Fiaf) or mTOR (Kim et al., 2010; Qiao et al., 2014; Jung et al., 2016), or by reversing the gut microbial dysbiosis caused by a high-fat diet by modifying the relative Bacteroidetes: Firmicutes ratio (Qiao et al., 2014; Sung et al., 2017). A recent study has shown that fecal microbiota transplantation from resveratrol treated mice to HFD mice reversed weight gain and improved gut microbiota composition and intestinal permeability (Wang et al., 2020). SIRT1 deficiency in the intestinal epithelium as studied with SIRT1 intestinal knock out mice, resulted in altered gut microbial composition, increased intestinal inflammation, and susceptibility to colitis implicating SIRT1 importance in maintaining intestinal tissue homeostasis through modulation of the gut microbiota (Wellman et al., 2017). In colonic biopsies from patients with inflammatory bowel disease (IBD), SIRT1 was downregulated by TNF-α and IL-21 in the mucosa (Caruso et al., 2014). Moreover, incubation with a SIRT1 agonist, Cay10591 reduced the acetylation of NF-κBp65 and suppressed the inflammatory cytokine production in the colon as seen in IBD. It also ameliorated experimental colitis induced in mice by reducing LPS-induced TNF-α production whereas a SIRT1 antagonist, Ex527 aggravated the same. On the contrary, another experimental study on mice and worms with an intestinal deletion of SIRT1 increased Paneth and goblet cell number and upregulated anti-bacterial peptides such as lysozyme and cryptidines resulting in a rearrangement of the gut microbiota, thus protecting them from colitis-induced colorectal cancer (Lo Sasso et al., 2014). The direct effects of resveratrol on SIRT1 or sirtuin activation in general is not completely conclusive as resveratrol as well as other sirtuin activators have many molecular targets that acts via diverse pathways on different sirtuin isoforms, depending on the substrate sequence and the type of acyl modification (Athar et al., 2009; Gertz et al., 2012; Britton et al., 2015; Gomes et al., 2019). With regards to SIRT1, resveratrol either directly binds and activates SIRT1 or increases the intracellular pool of NAD^+^ via phosphorylation of AMPK by serine-threonine liver kinase B1 (LKB1) or calcium/calmodulin kinase kinase-β (CaMKKβ) kinases on its catalytic α-subunit, that can be utilized by SIRT1 (Lan et al., 2017).

The aberrant microbiota to CNS pathway is thought to result in the formation of insoluble protein aggregates within neurons in neurodegenerative disorders (Quigley, 2017). Toxic accumulation of misfolded and aggregated α-synuclein protein, Lewy bodies, is seen in both CNS and ENS of Parkinson's disease (PD) patients (Beach et al., 2010). Gram-negative bacteria in these patients are abundant producing LPS which contributes to α-synuclein aggregation leading to dopaminergic neuronal death, thereby causing motor impairments through inflammatory pathways (Sharma and Nehru, 2015). Using mice that overexpress αSyn, it is shown that gut microbiota promotes motor deficits and microglia activation. αSyn aggregation results in progression of the disease (Sampson et al., 2016). Studies show the correlation between increased gut permeability due to endotoxin exposure and alpha-synuclein staining in early Parkinson's disease (Forsyth et al., 2011). PD is frequently associated with impaired gastric motility (Fasano et al., 2015). Several studies have supported the hypothesis that PD may initiate in the gut since GI dysfunctions appear many years before motor impairments suggesting spread of α-syn pathology from the ENS to the CNS. Alzheimer's disease (AD) is characterized by an accumulation of protein aggregates composed of amyloid-β peptide (Aβ) and tau in CNS tissues impairing cognitive function and the pathogenesis is believed to be associated with increased permeability of the gut and blood-brain barrierinduced by microbiota dysbiosis (Jiang C. et al., 2017). Gut bacteria can secrete large amounts of amyloids and LPS which modulates the signaling pathways that lead to neurodegeneration and AD pathogenesis, as well as inflammatory response to Aβ accumulation in CNS (Pistollato et al., 2016). Amyloid precursor protein (APP) from which Aβ is derived, is expressed in the ENS suggesting its role in the pathogenesis of AD (Arai et al., 1991). Studies with transgenic mice overexpressing APP has shown progressive accumulation of Aβ within enteric neurons leading to a decreased number of enteric neurons, dysmotility and intestinal inflammation (Semar et al., 2013; Puig et al., 2015), implying that ENS dysfunction could occur in AD.

The concept of microbiota-gut-brain axis is extensively studied, with an emphasis being on the gut dysbiosis in the onset and/or progression of several metabolic diseases such as obesity, type-2 diabetes mellitus, and the most commonly studied forms of neurodegeneration, such as AD and PD. This review examines scientific literature addressing the possible role of sirtuins in regulating this axis thereby targeting themselves as molecules of importance therapeutically. Each sirtuin has different targets, located in different subcellular locations, and might function quite differently. Therefore, it is extremely important to develop selective activators or inhibitors that target a specific sirtuin. Sirtuins are known to modulate gut microbiota. It is critical for future studies to clarify using sirtuins as interventions to correct dysbiosis, which may provide safe and effective treatments to slow or halt the progression of clinical disorders.

## Future Perspectives

This review offers a consolidated overview of sirtuins and their important functions in modulating neurodegeneration in the CNS and the ENS. The precise functions of sirtuins are still unclear, but they seem to be important players in age- and metabolism-associated neurodegeneration. Therefore, elucidating the molecular roles of sirtuins may enable the development of novel strategies for intervention in neurodegenerative diseases. Inhibition of SIRT2 while overexpressing SIRT1 is a potential strategy that could be used to treat certain age-related neurodegenerative diseases. The connection between sirtuins and dietary restriction also warrants further investigation on the precise role of sirtuins. However, the beneficial effect of dietary restriction on aging and various metabolic disorders is dependent on the activation of SIRT1 and can be mimicked by resveratrol. SIRT1 via resveratrol has shown neuroprotective effects against acute inflammation induced by colitis and are expressed by enteric neurons suggesting that it might help in gut motility and secretion. This could be a promising and previously unrecognized role of enteric sirtuins, especially SIRT1, in regulating energy homeostasis. Moreover, activation of enteric sirtuin pathways could offer a therapeutic approach to treating diabetes- and obesity-related gut dysfunction as well as age-induced neurodegeneration. Using genetically engineered reporter mice that illuminate the entire ENS (Jiang Y. et al., 2017), the effects of sirtuins on different neuronal subtypes can be better visualized and investigated when compared to traditional immunohistochemistry. Further research and identification of novel or repurposing of previously known small molecule activators and inhibitors of sirtuins could have a potential impact in the therapy of neurodegenerative disorders.

## Author Contributions

PC and SS designed the structure and contents of the review. PC, AV, and DA wrote and edited the manuscript. PC, AV, GL, SM, and SS provided critical revisions to the article. All authors contributed to the article and approved the submitted version.

## Conflict of Interest

The authors declare that the research was conducted in the absence of any commercial or financial relationships that could be construed as a potential conflict of interest.

## Abbreviations

Ab: Antibody
CR: Calorie restriction
CNS: Central nervous system
ENS: Enteric nervous system
GI: Gastrointestinal
HFD: High-fat diet
KO: Knock out
NDs: Neurodegenerative diseases
NF-κB: Nuclear factor kappa B
PGC-1α: Peroxisome proliferator-activated receptor gamma coactivator 1-alpha
ROS: Reactive oxygen species
Sirtuins: Silent information regulator genes: sirtuins
SCFA: Short chain fatty acid: SCFA
T2D: Type II diabetes
WT: Wild type.
